# Advancing and scaling up sexual and reproductive health services for climate adaptation and resilience in Uganda

**DOI:** 10.3389/fgwh.2025.1623785

**Published:** 2025-09-29

**Authors:** Niona Nakuya Kasekende, Charles Kabiswa, Joshua Zake, Monica K. Kansiime

**Affiliations:** 1 Regenerate Africa, Kampala, Uganda; 2 CABI, Nairobi, Kenya

**Keywords:** climate change, gender-based violence, policy, sexual and reproductive health, gender equality

## Abstract

**Background:**

Climate change significantly affects Uganda's economy and human well-being, with disproportionate impacts on the sexual and reproductive health (SRH) services of women and girls. However, SRH remains largely absent from climate change policy frameworks. This study examines the extent to which SRH is integrated into Uganda's climate policy frameworks and explores stakeholder perceptions of the impacts of climate change on SRH to provide insights for more inclusive and integrated policies.

**Methods:**

The study used a mixed-methods approach, combining policy document analysis with primary qualitative data. Key documents reviewed included climate change and health policies, strategies, plans, and national medium- and long-term development frameworks. Forty purposively selected key informants and 24 focus group discussions with 321 participants in Buikwe District were conducted. Qualitative data were analyzed using content analysis to identify key themes and gaps.

**Results:**

Uganda's climate policy frameworks acknowledge the gender-differentiated impacts of climate change and highlight the importance of SRH services but fall short of outlining concrete actions to address SRH within climate adaptation and mitigation strategies. Key informants highlighted limited stakeholder awareness and weak institutional coordination as major barriers to integrating SRH into climate action. Community respondents noted that climate extremes degrade critical infrastructure, disrupt access to SRH services and increase vulnerabilities, including a heightened risk of gender-based violence.

**Conclusions:**

The impact of the climate crisis on SRH is increasingly evident, particularly for women and girls, yet Uganda's key climate policies still exhibit lack of concrete actions to address SRH vulnerabilities. Prioritizing SRH within climate adaptation efforts, especially through resilient health systems and livelihood support such as climate resilient agricultural training and vocational programs for women and girls is key to advancing both gender and health equity, and climate resilience in Uganda. This should be supported by robust gender disaggregated data, stronger institutional coordination, and inclusive, community-led planning.

## 1 Background

Uganda is one of the most climate-vulnerable countries in the world, ranked 13th (1) due to its heavy reliance on climate-sensitive sectors such as agriculture, fisheries, and forestry (2, 3). Agriculture alone contributes 24% of Uganda's gross domestic product (GDP) (4). Agriculture is also the primary source of employment, engaging 72% of the country's labor force, especially in the rural areas (5). Yet the sector remains heavily reliant on rainfed practices, with less than 2% of the land area under irrigation (6). This dependence on natural rainfall makes Uganda highly susceptible to climate-related shocks such as erratic rainfall, prolonged droughts, and extreme weather events disrupts agricultural productivity, reduces fish stocks in water bodies, and exacerbates land degradation and biodiversity loss, further deepening poverty and livelihood insecurities.

Climate-related disruptions have cascading effects on health, especially sexual and reproductive health (SRH). Extreme weather events such as floods damage health infrastructure, disrupt service delivery, and limit access to essential SRH services—including contraceptives, maternal and child healthcare, STI prevention and treatment, and menstrual hygiene support (7). Climate change has also contributed to the rise of vector-borne diseases; for example, malaria infections among highland residents in Uganda increased from 3% in 2010 to 12% in 2020 due to warmer temperatures and increased rainfall (8). Beyond these direct effects, climate change triggers secondary impacts through the erosion of livelihoods. Reduced household incomes limit the ability to afford SRH services, especially in rural and underserved communities. Food insecurity intensifies these challenges by driving harmful coping strategies such as early marriage and transactional sex, which increase the risk of unintended pregnancies, sexually transmitted infections, and gender-based violence (9, 10). At the systemic level, economic strain weakens public financing for health infrastructure and SRH programs, further compounding access and equity challenges.

Conversely, SRH is a critical yet under recognized climate change adaptation strategy. Ensuring access to comprehensive SRH services not only upholds fundamental human rights but also strengthens the adaptive capacity of individuals and communities, particularly women and girls (11, 12). Access to SRH services enables women to make informed reproductive choices, which supports educational attainment and economic participation—both of which are essential for household and community resilience in the face of climate shocks. Integrating SRH into climate-resilient health systems—including mobile outreach, supply chain management, and emergency preparedness—ensures continuity of care during disruptions, reinforcing health system resilience. As the linkages between SRH and climate change become more established, it is important to understand how they may be accounted for in climate change planning, policy, and programming.

Uganda has various climate change policy frameworks such as the National Climate Change Policy (2015), the National Adaptation Programme of Action (2007), the Health National Adaption Plan (2025–2030), and the recently approved 4th National Development Plan (2025/26–2029/30) that emphasize cross-sectoral collaboration. In 2015, Uganda adopted the Paris Agreement, along with other 196 parties, and has developed Nationally Determined Contributions to tackle climate change. This international treaty that aims to strengthen countries' resilience to the impacts of climate change and support them in their adaptation efforts, highlights the need for gender-responsive climate action. Uganda's Vision 2040 is aimed at supporting long-term economic and social development. National climate policies and regional commitments are critical in shaping responses to climate change, particularly in building adaptive capacity and resilience. However, considerable gaps remain in understanding how these frameworks address the intersection of climate change and SRH.

This study aimed to: 1) examine the institutional integration of SRH in Uganda's climate policy framework and identify key policy gaps;; 2) explore stakeholder perceptions of the gendered impacts of climate change on SRH; and 3) generate actionable insights to strengthen SRH integration in climate adaptation strategies that address both environmental and social challenges. This study enhances understanding of existing policy approaches and highlights key areas for improvement and greater inclusion. Strengthening the integration of SRH in climate policies can ensure more comprehensive, equitable, and gender-responsive climate adaptation strategies, that are scalable to local communities.

## 2 Conceptual framework

**Figure 1:**
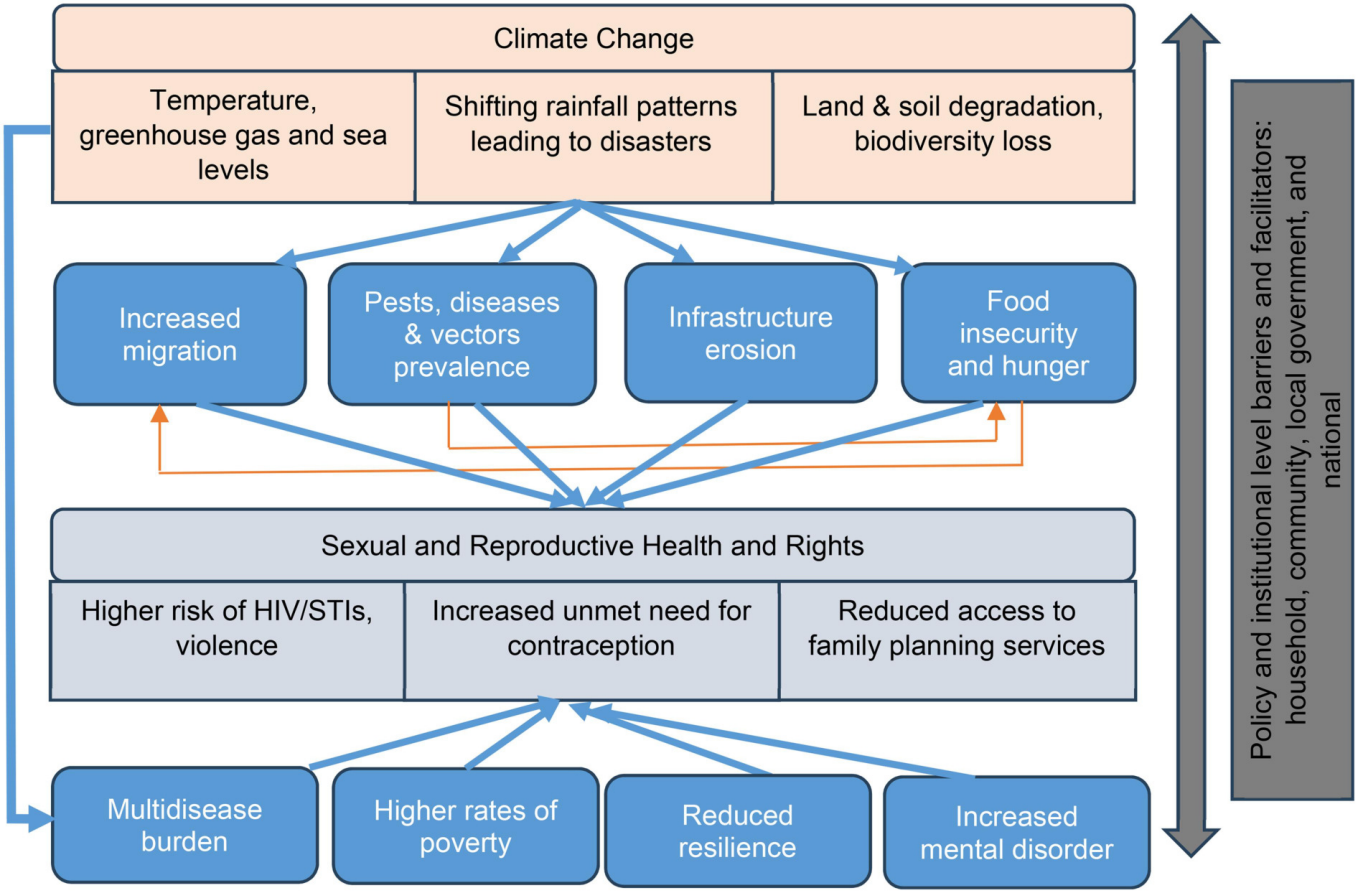
Conceptual frameworks linking climate change with SRH outcomes. Adapted from (7).

Empirical evidence shows four pathways linking climate change with SRH (Figure 1). The conceptual framework shows that various dimensions of climate change such as rising temperatures; shifting rainfall patterns (floods, unreliable rainfall, prolonged droughts); and land and soil degradation, impact SRH. This is through four pathways: increased human migration; increased prevalence of pests, diseases and vectors; erosion of public health infrastructure; and increased food insecurity and hunger.

Based on this framework, our study proposes that these pathways impact SRH outcomes, as they have a direct impact on adaptive potential of individuals, households, and communities. For instance, climate change-induced migration due to resource scarcity (water, firewood, grazing land, food) increases SRH challenges by increasing exposure to sexual violence and exploitation, disrupting access to healthcare, heightening risks of unplanned pregnancies and STI and HIV transmission (13). The breakdown of healthcare infrastructure limits access to maternal care, contraception, and menstrual hygiene resources, while economic hardships drive early and forced marriages, further endangering adolescent girls.

Food insecurity heightens vulnerabilities to transactional sex, child marriage, and gender-based violence, as individuals—especially young girls—may be forced into exploitative relationships in exchange for food and resources. Additionally, the stress and anxiety associated with food insecurity can lead to poor mental health, affecting reproductive decision-making and access to contraception. Increased crop pests and livestock diseases threaten food security and household incomes, exacerbating malnutrition, particularly among pregnant women and children, leading to higher risks of maternal and neonatal complications. The spread of vector-borne diseases like malaria, which are intensified by changing climatic conditions, disproportionately affects pregnant women, increasing the likelihood of miscarriage, stillbirth, and low birth weight.

Realization of SRH, particularly for those already facing discrimination can empower people to exercise their agency and engage in climate action. For those affected by intersecting inequalities, the realization of SRH can allow the needs and priorities of marginalized groups to be better addressed including in climate action and policy. Realization of SRH is fundamental to achieving gender equality, making it a key aspect of gender-responsive climate action.

The conceptual framework guided the development of tools and subsequently the organization of our research results. Key questions focused on understanding community and local leader perception of climate change and trends over time, and how these factors directly or indirectly affect socio-economic status of communities, and subsequently access to SRH services, including risk of gender-based violence, STIs, contraception and family planning services. Questions also covered how policy and institutional frameworks integrate actions to address SRH at national, regional and community levels.

## 3 Methods

### 3.1 Study location

The study focused on climate actions and SRH services at the national and local levels. At the local level, data were obtained from Ssi-Bukunja Sub-County, Buikwe District. The district is located within the Lake Victoria Basin (LVB), one of Africa's most densely populated regions, with over 200 people per square kilometer. The region is also characterized by rapid population growth and high levels of poverty. The basin's communities depend heavily on natural resources such as water, wetlands, forests, and fish for their livelihoods, with agriculture and livestock being dominant sectors. Fishing also provides a direct livelihood for over 800,000 individuals in the broader region. Yet, the region faces intense environmental degradation due to factors like rapid population growth, agricultural expansion, and climate change. These pressures have led to the loss of natural habitats, including wetlands and forests, which negatively affect terrestrial biodiversity. These issues highlight the region's vulnerability to both climate variability and population growth, making it a critical location for studying the intersection of climate change and SRH.

### 3.2 Study design

The study employed an in-depth analytical research design and was conducted in two phases. In the first phase, data were collected using policy content analysis. In the second phase, primary data were gathered through key informant interviews and focus group discussions.

#### 3.2.1 Policy content analysis

A policy content analysis was conducted to identify gaps and opportunities for integrating SRH and related thematic areas within Uganda's climate change policies. The review focused on national climate change initiatives, including climate action plans, policies, strategies, and the country's medium- and long-term development plans. Table 1 provides an overview of the policy frameworks analyzed in this study.

**Table 1:** Reviewed climate change policy frameworks.

Policy framework	Year	Type of policy
1. Health National Adaptation Plan	2025	Policy
2. Nationally Determined Contributions (NDCs)	2022	Plan
3. Uganda National Climate Change Policy	2015	Policy
4. The Uganda Green Growth Development Strategy 2017/18–2030/31	2017	Strategy
5. National Population, Health and Environment Network Strategic Plan 2020/21–2024/25	2020	Strategy
6. National Policy for Disaster Preparedness & Management	2011	Policy
7. Uganda Vision 2040	2007	Long term plan
8. Fourth National Development Plan (NDPIV) (2025–2030)	2025	Medium-term plan

#### 3.2.2 Primary data collection

Qualitative data collection approaches were used purposively targeting local-level participants and key informants at both the local and national levels.

##### 3.2.2.1 Focus group discussions

Focus group discussions (FGDs) were conducted to gather community perceptions and opinions with respect to the linkage between SRH and climate actions. The FGDs comprised participants representing various sectors, genders, and age groups. Twenty-four FGDs involving a total of 321 community representatives (109 adult women and 122 adult men; 49 young men and 41 young women) were conducted in 6 villages of Musomoko, Gaba landing site, Nalumuli, Ssenyi, Kiwunya, and Lukubo. Young men and women were aged between 18 and 25 years. The FGDs explored community perception of climate change and its impacts on livelihoods, access to SRH, and other health services.

##### 3.2.2.2 Key informant interviews

Key informants were interviewed to obtain their perceptions with respect to existing actions implemented by stakeholders, institutional barriers, best practices, and expertise in advancing and scaling up SRH for climate action in Uganda. The key informants were selected purposively with due consideration that they are actively involved and have expertise in sexual and reproductive health, gender dynamics, or climate change resilience and adaptation at the policy and practice level. Overall, 40 key informants (22 women and 18 men) participated in the study. They included senior and middle level policy makers from public sector (Ministry of Health; Ministry of Water and Environment; Ministry of Finance, Planning and Economic Development; National Population Council; Ministry of Gender and Social Development; Ministry of Local Government), representatives from professional associations and non-state actors (National PHE Network, Civil Society Organizations, Community-based Organizations); community leaders, community health workers, and women's groups, Village Health Teams, and Beach Management Units.

Key informant interviews explored the major climate change events and impacts in Uganda and Buikwe District, assessing their effects on SRH and overall health service delivery. Respondents were asked to highlight the specific challenges faced by healthcare providers, district local government, and central government ministries in delivering these services amid climate-related disruptions, as well as opportunities for integrating SRH into climate adaptation and resilience efforts.

### 3.3 Data analysis and synthesis

Policy content analysis involved a systematic review of selected documents to assess how sexual and reproductive health (SRH) and social inclusion were addressed within climate change policies. This entailed examining both the frequency and contextual use of key SRH-related terms—such as sexual health, family planning, contraceptives, maternal and newborn health, gender, women, girls, mothers, inclusiveness, equity, and equality. In addition, the analysis identified references to broader social markers, including youth, age group, poverty, marginalization, socio-economic status, smallholder farmers, ethnicity, and ethnic groups, to evaluate how well the policies considered intersectional vulnerabilities and promoted inclusive climate responses. Complementary data from key informant interviews and focus group discussions (FGDs) were analyzed using qualitative content analysis to enrich and triangulate findings from the policy review.

Analytical depth and data richness were ensured through several deliberate strategies. First, purposive sampling was used to capture diverse perspectives across different stakeholder groups, ensuring broad representation and thematic saturation. Data collection tools—including key informant interview guides and focus group discussion protocols—were semi-structured, allowing for both consistent inquiry and flexibility to probe for in-depth responses. With informed consent, all interviews and FGDs were digitally recorded to ensure accuracy and completeness of responses. These recordings were later transcribed verbatim. Where discussions or interviews were conducted in local languages, professional translation into English was undertaken. This ensured that local expressions, meanings, and cultural nuances were accurately retained and integrated into the dataset. Triangulation of data sources—including policy document reviews, key informant interviews, and FGDs—further enhanced analytical rigor, allowing for validation and enrichment of findings across methods.

### 3.4 Research ethics considerations

The study was conducted as part of an ongoing project activity, in collaboration with local leaders and community health workers to ensure adherence to locally accepted socio-cultural, safeguarding and ethical standards. The research team obtained informed consent from the participants before any information was recorded. First, they explained the purpose of the study, the process and duration of the inquiry, as well as how participants' privacy would be kept. This includes keeping the respondent's identity and personal information confidential and private, and data restricted for access to only the research team and not to be disclosed in any publications and presentations. Although the study did not seek formal approval from an institutional review board (IRB), all efforts were made to uphold ethical research principles throughout the process.

## 4 Results

### 4.1 Climate change policy frameworks and how they integrate SRH

A systematic content analysis of Uganda's climate change policy frameworks reveals that while the country has developed several institutional mechanisms to address climate change through health, gender, and resilience-focused policies, SRH remains largely absent from climate adaptation and mitigation strategies. For example, while Uganda's NDCs emphasize gender-responsive climate measures and health sector resilience, they do not explicitly incorporate SRH services such as family planning or maternal and newborn health. Similarly, the Uganda National Climate Change Policy briefly references family planning and reproductive rights but lacks concrete policy interventions or operational frameworks to address these aspects. The National Disaster Preparedness Policy acknowledges gender disparities in climate impact vulnerability but omits direct references to SRH, highlighting a policy gap in integrating reproductive health considerations within disaster preparedness and climate resilience planning.

Among the reviewed policies, the Population, Health, and Environment (PHE) Strategy presents a more integrated approach, explicitly recognizing family planning and reproductive health as essential for sustainable natural resource management. This strategy advocates for gender mainstreaming and emphasizes the interconnections between population dynamics, health, and environmental sustainability. However, despite its integrative nature, its implementation remains limited, and its influence on climate change policies is minimal.

Broader national development frameworks, including Uganda Vision 2040 and the National Development Plans (NDPs) acknowledge health as a driver of socio-economic transformation. Whereas the NDP III does not explicitly integrate reproductive maternal and newborn health in its interventions, the NDP IV proposes to increase access to SRH information and services and improve maternal, adolescent and child health services. However, this is not directly aligned or integrated with climate adaptation and mitigation efforts despite the recognized vulnerability of the country to climate-related risks.

Uganda's Health—National Adaptation Plan (H-NAP) is a strategic framework designed to enhance the resilience of the health sector against climate change by systematically integrating climate considerations into health policies, programs, and planning. Its components and activities are inclusive of SRH and aim at strengthening climate-resilient health systems, enhancing early warning systems and response to climate-sensitive illnesses, promoting climate-informed health programs, and mobilizing sustainable financing for health and climate interventions. However, SRH does not have standalone activities but is integrated with other aspects like WASH, nutrition, air pollution, etc. which might make it difficult to track.

The overall policy landscape reflects a fragmented approach to integrating SRH into climate change policies. Table 2 shows a summary of the reviewed policy frameworks, identifying strengths and gaps for effective integration of SRH in climate change policies.

**Table 2:** Integration of SHRH in climate change policies and frameworks in Uganda.

Policy framework	Purpose	Integration of SRH and related thematic areas	Gaps
Health National Adaptation Plan, 2025–2030 (14)	To establish a responsive health system that promotes inclusive Climate Change Adaptation measures.	SRH has been integrated under H-NAP components such as management of the environmental determinants of health; health and climate research; climate informed health programming.	No standalone activities for SRH but rather SRH is conjoined with other aspects like WASH, air quality, and nutrition.
Updated Nationally Determined Contributions (15)	NDCs are national climate action plans under the Paris Agreement. The NDC outlines how Uganda plans to reduce greenhouse gas emissions to help meet the global goal of limiting temperature rise to 1.5C and adapt to the impacts of climate change.	Acknowledges population dynamics, particularly migration, in climate change. Highlights reproductive health and building a resilient health sector. NDC integrates gender sensitivity in measures and actions, emphasizing the need for gender-responsive implementation.	No specific mention of aspects of family planning and maternal and newborn health.
Uganda National Climate Change Policy (34)	The policy provides direction to all sectors that are affected by climate change to facilitate adaptation and mitigation and to strengthen coordination of efforts amongst all sectors to build an overarching national development process that is more resilient.	Acknowledges the link between population growth and GHG emissions, advocating FP, MNH and RH. Notes climate change impacts on sectors, emphasizing gender-sensitive response policies.	Topics on family planning, maternal and newborn health and reproductive health are not detailed but only mentioned in passing.
The Uganda Green Growth Development Strategy 2017/18–2030/31 (16, 17)	It serves as a framework and/or guidance tool that aims at catalyzing economic growth through the efficient use of the country's natural, human, and physical capital in an inclusive manner along a low emissions development pathway.	Recognizes health as a crosscutting issue which is affected by climate change and can be improved when integrated with climate change efforts.	There is no mention of SRH.
National Population, Health and Environment Network Strategic Plan 2020/21–2024/25 (35)	This Plan is a roadmap that lays down specific priorities to guide population, health and environment (PHE) interventions in the country. The goal is to contribute to national development through the integration of PHE into Government policies, plans and programmers.	Mentions using the integrated PHE approach to address environmental pressures, population mobility, and refugee challenges. Identifies reproductive health and family planning as key livelihood activities, prioritizing health for sustainable natural resource management. Advocates gender mainstreaming in implementation to address reproductive health issues.	None
The National Policy for Disaster Preparedness and Management (36)	The policy aims to promote national vulnerability assessment, risk mitigation, disaster prevention, preparedness, effective response and recovery in a manner that integrates disaster risk management with development planning and programming.	Mentions population dynamics and health issues. Recognizes that women, children, the elderly, and persons with disabilities are more affected by disasters.	No specific mention of SRH, and related issues.
The Uganda Vision 2040 (18)	The document provides development paths and strategies to aimed at transforming Uganda from a predominantly peasant and low-income country to a competitive upper middle-income country.	Recognizes that health is instrumental in facilitating socio-economic transformation and the role of reproductive health in improving the quality of the population.	There is no specific reference to maternal and newborn health and gender equity.
The Fourth National Development Plan (NDPIV) 2025/26–2029/30) (17)	The plan aims to achieve higher household incomes, full monetization of the economy, and employment for sustainable socio-economic transformation.	It calls for equitable access to SRH services and information, highlighting specific components of SRH like maternal, adolescent and child health.	The plan does not explicitly highlight the linkages between health and climate and hence responsive interventions can only be derived.

### 4.2 Local stakeholder perception of climate risks and impacts

Focus group discussions (FGDs) with community members revealed significant climate-related events over the past 40 years. The key climate hazards reported by the communities was variations in rainfall. This variation often leads to floods triggered by heavy rainfall, followed by periods of prolonged droughts and water scarcity, directly affecting agricultural production and contributing to destruction of infrastructure and reducing access to health, education and other services.

In terms of agricultural production, both women and men reported that extreme weather conditions have significantly reduced agricultural yields, led to livestock deaths and poor fish catches, leading to food shortages and loss of income. According to respondents, these conditions have resulted in hunger and malnutrition, particularly affecting children and pregnant women. Participants in focus group discussions recalled the severe drought and famine of 1987, as well as intermittent flooding along the lakeshores between 2019 and 2020, followed by a prolonged drought in 2022. These episodes led to poor yields and in some cases total crop loss, leading to widespread food insecurity. Poor rainfall patterns also affect vegetation and pasture abundance, creating scarcity of pasture and fuel wood. This in turn has placed more pressure on natural resources and particularly driven encroachment of wetlands and environmentally sensitive areas. Respondents also highlighted that too much rainfall and water stagnation has promoted proliferation of vectors in their communities increasing incidence of vector-bone sicknesses such as malaria and intestinal worm infections.

In Gaba landing site, respondents reported experiencing intense rainfall accompanied by strong winds in 2021, leading to extensive damage to homes, infrastructure, farmlands, and a local school. These conditions also resulted in the rising water levels in Lake Victoria causing shoreline flooding and disrupting fishing activities, a primary source of livelihood for many men in the area. Similarly, in Nalumuli village, fishermen highlighted the increasing frequency of strong winds that often-capsized boats, making fishing highly unpredictable and hazardous. The reduction in safe fishing days has had a cascading effect on income, food security, and household resilience.

Respondents cited the destruction of Buikwe Health Center II in 2019 due to floods, leading to the destruction of medical supplies and important documentation. The facility, which served over 200 patients and provided a wide range of essential services—including inpatient and outpatient care, antenatal and postnatal services, family planning, laboratory testing, child immunization, community outreaches, counselling, and HIV screening—was rendered non-operational. As a result, access to these critical healthcare services was significantly disrupted, impacting the well-being of the community.

**Figure 2:**
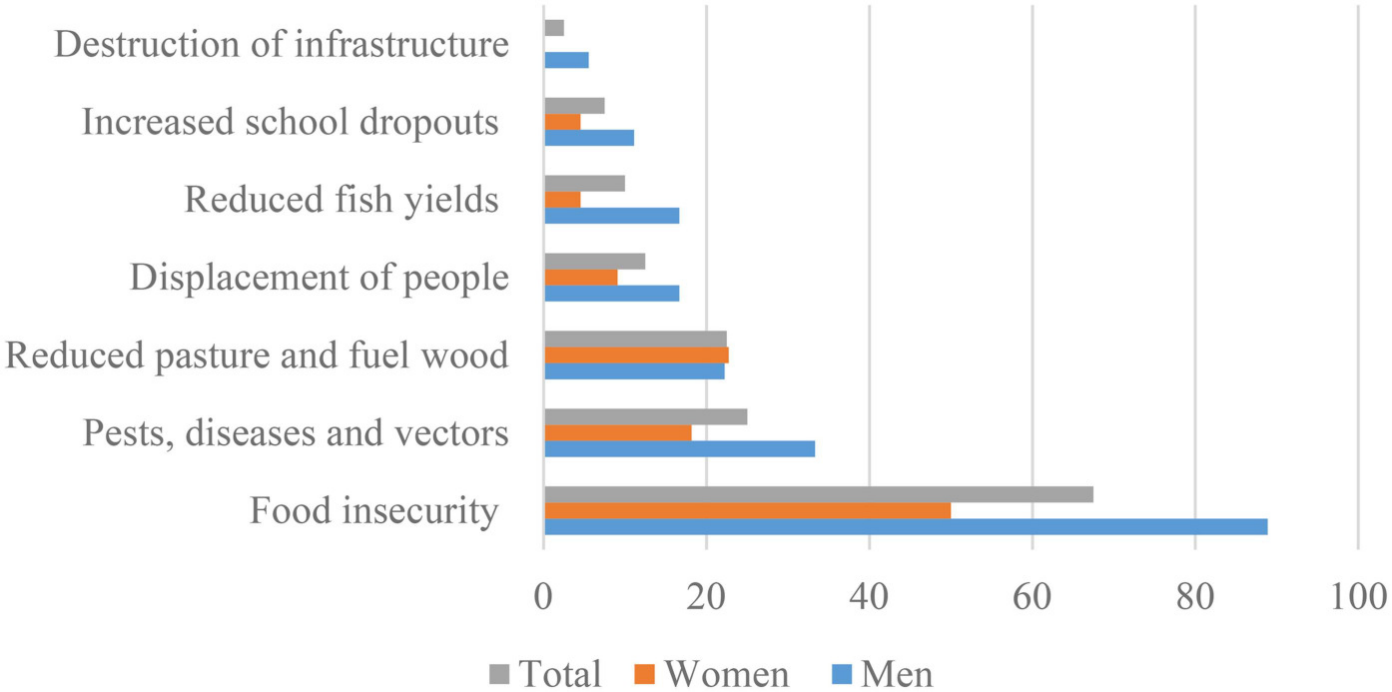
Climate impacts as reported by key informants at local and national levels (*n* = 40).

Responses from focus group discussion were in tandem with those from key informants. Key informants prioritized food insecurity as the key direct impact of climate change in Uganda as a whole, citing hunger-related deaths in some parts of the country (Figure 2). Key informants also mentioned increased pests, diseases and vectors which have led to crop loss, and for human's increase in water related diseases. Reduction in pasture and fuel wood, and displacement of people due to floods and mudslides has led to resource conflicts.

### 4.3 Local stakeholder perceptions of climate change and linkage with SRH

This section presents qualitative findings from focus group discussions and key informant interviews on how climate change affects access to SRH services in Buikwe District. Drawing on lived experiences shared by community members and health workers, the analysis is organized thematically to highlight four key areas of impact: physical access to services, disruption of healthcare infrastructure, psychosocial consequences such as gender-based violence, and gendered coping strategies in response to climate-induced hardship.

#### 4.3.1 Physical access

Respondents noted that climate-induced flooding frequently renders roads impassable, significantly restricting access to already distant healthcare facilities, some located 10–15 kilometers away. These mobility constraints also delay medical referrals, which are critical for emergency management, thereby increasing the risk of maternal and neonatal mortality. In addition, adverse weather conditions—such as heavy rainfall and extreme heat—discourage women from making the long and often physically demanding journey to health facilities. One female respondent described the challenge:

“I often feel discouraged from going to the health facility because of the long journey I have to make. It takes an entire day just to walk there, receive the necessary services, and return home. This frustration intensifies during extreme weather—whether heavy rains or scorching heat—making it even harder to leave the house on such days.”

Disruptions in mobility due to climate-induced environmental changes have significantly impacted school attendance, with community members reporting an increase in dropout rates. This trend is particularly pronounced among adolescent girls, who face heightened risks of early marriage and exposure to sexually transmitted infections (STIs) upon leaving school. The loss of educational opportunities not only limits their future economic prospects but also exacerbates vulnerabilities related to health and social well-being.

#### 4.3.2 Service disruption

Recurrent flooding has also caused significant damage to healthcare infrastructure. For example, respondents recalled severe flooding events that impacted Buikwe Health Centre III in 2019 and Namwezi Health Centre III in 2020, compromising the availability of essential maternal and reproductive health services. Additionally, climate-induced displacement and migration further disrupt access to SRH and general health services, with some individuals missing out on critical care altogether. The health impacts of climate change extend to sanitation, with recurrent flooding leading to the collapse of pit latrines, overflowing sanitary pits, and waterlogged garbage disposal sites. Women and young people reported that poor access to sanitation facilities negatively affected menstrual hygiene, while climate-induced poverty further limited access to menstrual hygiene products such as sanitary towels. Health workers in the region have raised concerns that the increasing frequency of such climate-related health crises underscores the urgent need for improved water, sanitation, and hygiene (WASH) infrastructure to mitigate these impacts.

#### 4.3.3 Psychosocial impacts

The adverse effects of extreme weather events extend beyond service disruption, exacerbating food insecurity and economic instability. These conditions have driven some households to resort to early marriage as a financial coping mechanism, a practice that further entrenches cycles of poverty and gender inequality. Concurrently, financial strain within households has been strongly associated with increased incidences of gender-based violence, including verbal, physical, and sexual abuse. Male respondents in Nalumuli emphasized the link between economic hardship and domestic violence, stating:

“When men cannot be the breadwinners as they are expected to be, they become stressed, leading to ‘a recipe’ for violence.”

Respondents indicated that women experiencing domestic violence feel discouraged from seeking SRH services, including family planning, particularly when male partners oppose their use. Additionally, qualitative findings from Musomoko and Lukubo villages indicate that during climate crises, women tend to priorities food security over access to SRH services. Such shifts in resource allocation and decision-making negatively impact health-seeking behaviors, leading to increased risks of unintended pregnancies, maternal and neonatal morbidity, and limited autonomy over reproductive choices.

Respondents also reported an increased risk of HIV and STIs due to transactional sex, exacerbated by poverty-driven limitations in access to protective services. Additionally, inadequate nutrition weakens immune systems, reducing the effectiveness of antiretroviral therapy and increasing susceptibility to opportunistic infections.

#### 4.3.4 Gendered coping strategies

Women and girls respondents indicated that they are disproportionately affected by climate change due to their roles as primary caregivers and environmental custodians. Their responsibilities including securing food, water, and firewood have become more burdensome during extreme weather events. Water scarcity, in particular, affects girls' menstrual hygiene, leading to school absenteeism. Additionally, the increasing distance and time required to fetch water add to the physical burden on both boys and girls.

Women's heavy involvement in agriculture makes them more vulnerable to climate-related livelihood loss, further exacerbating gender inequalities and limiting their contributions to community development. One female key informant highlighted the intersection of climate-induced economic hardship and SRH challenges:

“Women are among the most affected by extreme weather events, particularly due to limited access to family planning services. This often results in unplanned pregnancies at a time when they are already grappling with loss of property and economic hardship. Facing a disaster makes it incredibly challenging to care for a pregnancy, let alone provide for children, further increasing their vulnerability.”

These results highlight the multifaceted ways in which climate change exacerbates vulnerabilities in sexual and reproductive health, gender-based violence, and socio-economic well-being.

### 4.4 Barriers to the integration of SRH in climate actions

Key informants at both national and local levels identified several structural, financial, and socio-cultural barriers hindering the effective integration of SRH into climate actions. These barriers are multifaceted, affecting policy coherence, program implementation, and service delivery. The key barriers have been categorized into structural and cultural.

#### 4.4.1 Structural barriers

1. **Resource constraints:** A primary constraint identified was the insufficient financial, human and technological resources within the government ministries and agencies which limit the integration of SRH services into climate adaptation programs. Climate-related emergencies, such as droughts and floods, receive priority, leading to chronic underfunding of reproductive health initiatives.
2. **Lack of a coordination mechanism:** The absence of a centralized oversight body results in fragmented and inefficient interventions, with frequent duplication of efforts. Without a dedicated coordinating entity, climate adaptation programs fail to systematically incorporate SRH components.
3. **Institutional silos and policy fragmentation:** Rigid institutional mandates restrict cross-sectoral collaboration, leading to isolated program implementation. Policy frameworks often address climate change and reproductive health separately, lacking integration to reflect their interdependencies. Without coherent policies, efforts to link SRH and climate adaptation remain disjointed and ineffective.
4. **Limited knowledge and technical expertise:** A recurring theme in the discussions was the insufficient technical expertise among policymakers and program implementers to integrate SRH and climate resilience effectively. Informants noted that many decision-makers lack the necessary knowledge and skills to establish practical linkages between gender, reproductive health, and climate adaptation.
5. **Data gaps:** The absence of reliable, disaggregated data impedes evidence-based decision-making. Without comprehensive data on climate vulnerabilities and reproductive health needs, targeted interventions are difficult to design and implement. Strengthening data collection mechanisms and incorporating SRH indicators into climate assessments are critical for policy and program development.

### 4.42 Cultural barriers

1. **Economic constraints and competing priorities at household level:** Poverty remains a fundamental barrier to both climate resilience and SRH access. Informants noted that individuals and communities facing economic hardship priorities immediate survival needs over long-term investments in reproductive health and climate adaptation. Addressing economic constraints through livelihood support, microfinance, and social safety nets can significantly improve access to SRH services within climate-vulnerable populations.
2. **Socio-cultural and religious barriers:** Cultural and religious beliefs continue to pose substantial resistance to SRH integration, particularly in rural and conservative communities. Key informants highlighted that family planning and reproductive autonomy are often viewed as Western impositions, leading to reluctance in adopting SRH interventions. Informants emphasized the need for community-led advocacy strategies that engage religious and traditional leaders to facilitate behavior change and foster acceptance of SRH services within climate adaptation initiatives.

## 5 Discussion

The findings reveal that Uganda's policy environment acknowledges the intersections of health, gender, and climate change, but SRH is insufficiently mainstreamed across climate policies. Existing references to family planning, maternal and newborn health, and reproductive rights remain broad and lack operational detail. Where SRH is included—as in the PHE Strategy and H-NAP—implementation challenges and integration within broader climate frameworks limit impact. A major barrier lies in institutional fragmentation: SRH is primarily managed under the Human Development Programme, while climate action falls under the Natural Resources, Environment, Climate Change, Land, and Water Management Programme. These silos restrict cross-sectoral collaboration, preventing comprehensive approaches to interconnected challenges. Additionally, stakeholder awareness of SRH–climate linkages remains low, reducing opportunities for coordinated advocacy and programming (13). Moving forward, embedding SRH considerations within Uganda's climate policies will require deliberate multi-sectoral collaboration, strengthened institutional capacity, and more coherent policy design. Evidence-based advocacy can help position SRH as a core component of resilience and adaptation strategies, while ensuring gender equality commitments are fully operationalized. Greater attention to tracking indicators specific to SRH within climate frameworks will also be essential for accountability and progress monitoring. The study focused on eight key climate policies, offering in-depth insights into how SRH is integrated within these frameworks. While it did not explore references to climate in other sectoral policies or examine SRH integration from the reverse perspective, this provides a strong foundation for future research. Similarly, although cross-policy comparisons were beyond the scope of this study, the findings highlight important entry points for more comprehensive, comparative analyses going forward.

The perspectives of communities and key informants align closely with broader climate trends observed in Uganda and the Lake Victoria Basin where increasing climate variability has exacerbated vulnerabilities in food security, income, and health outcomes. Within the Lake Victoria basin, the annual occurrence of devastating floods in the region during the October-December rainy season, leading to significant loss of life and property. This was corroborated using time series and spatial analyses (19). Warming water temperatures, modified hydrological processes, increased pollutants and occasional flooding have altered fish distribution and abundance, negatively impacted livelihoods of fishery-dependent people (20, 21).

At the national level, document rising temperatures, heightened rainfall variability, and more frequent extreme weather events over the past three decades (22). Research further indicates that unpredictable rainfall patterns and prolonged droughts have increased contributing to declining agricultural productivity, heightened pest infestations, and a surge in crop diseases (23). The adverse effects of climate variability are particularly evident in the Karamoja sub-region of northeastern Uganda, where prolonged droughts have led to severe food insecurity, resulting in over 900 hunger-related deaths in 2022 alone (24). Additionally, studies by (25, 26)highlight the escalating burden of waterborne diseases—including dysentery, cholera, hepatitis E, and malaria—alongside increased cases of respiratory infections and malnutrition-related illnesses due to climate change. These findings underscore the critical need for targeted adaptation strategies to enhance resilience among vulnerable communities in the region.

The local stakeholder perceptions of climate change and its linkage with SRH highlight the complex ways in which climate change intensifies vulnerabilities in SRH access, gender-based violence, and socio-economic well-being. Findings reveal that climate shocks act as both direct barriers to service access (e.g., damaged infrastructure, mobility challenges) and indirect drivers of negative health and social outcomes (e.g., early marriage, transactional sex, and intimate partner violence). Other research has shown that more women resorted to home births without skilled attendance—a well-documented risk factor for maternal and neonatal morbidity and mortality in resource-constrained settings (27, 28). As evidenced in previous research, economic distress drives families to adopt harmful coping strategies, including child marriage and transactional sex (29). Additionally, economic hardship and environmental stressors contribute to intimate partner violence, reinforcing gender inequalities and limiting women's access to essential health services (30). The compounding effects of economic insecurity, gendered social norms, and environmental stressors underscore the urgent need for integrated policies that address both climate resilience and equitable access to healthcare services. Strengthening WASH systems, safeguarding healthcare infrastructure, and embedding SRH into climate adaptation frameworks are critical steps. Moreover, gender-responsive adaptation strategies must go beyond service provision to tackle structural inequalities, empower women and girls, and protect them from harmful coping strategies that arise in the face of climate-induced hardship.

Sentiments from key informants on the barriers to the integration of SRH in climate actions align with empirical evidence which highlights that inadequate health financing in many low-income countries continues to impede access to essential services. World Bank highlights that over half of the world's population lack access to essential health services, and nearly 2 billion people face financial hardship due to the high costs of health care (31). Increased out-of-pocket health spending could push people further into poverty, affecting achievement of sustainable development goals. Similarly, the need for multi-sectoral coordination is emphasized which underscores that well-structured coordination mechanisms enhance policy coherence, optimize resource allocation, and improve the efficiency of integrated programs (32,33). Effective collaboration across sectors is essential for addressing the complex intersection of climate change, health, and gender disparities. This coordination should be supported for sufficient capacity building and data-driven approaches to ensure effective integration of SRH in climate change actions (22). Without targeted training and technical support, opportunities for meaningful policy and programmatic integration remain limited. Collectively, findings from this study and empirical evidence highlight the necessity of a holistic, well-resourced approach to integrating SRH within climate adaptation and social protection frameworks.

Overall the study focused on one geographical location—Lake Victoria basin—providing valuable insights into the local context. However, this focus limited opportunities for broader disaggregation by gender, age, or socio-economic status, highlighting the need for future research across multiple regions and climate contexts to capture more diverse experiences.

## 6 Conclusions and recommendations

### 6.1 Conclusions

This study shows that while current policy frameworks in Uganda acknowledge gendered vulnerabilities due to climate change, particularly affecting women's sexual and reproductive health rights (SRH), they lack comprehensive implementation mechanisms and strategies to ensure that SRH is integrated within climate adaptation and mitigation strategies. The lack of disaggregated data by gender, age, and socio-economic status limits policymakers' ability to design targeted interventions. Key informants and community representatives emphasized that climate change significantly worsens SRH disparities, disproportionately impacting women and girls. These effects manifest through increased economic vulnerabilities, restricted access to healthcare, and heightened exposure to gender-based violence. Empirical evidence confirms that climate change impacts are not gender-neutral, with women and children being among the most at-risk groups. Several factors contribute to the heightened vulnerability of women compared to men, including gender-based disparities in time use, unequal access to assets and credit, and limited participation in policy processes and decision-making. These structural inequalities hinder women's adaptive capacity, further exacerbating their susceptibility to climate-related risks.

### 6.2 Recommendations

At the national level, the Ministry of Water and Environment, or the Ministry of Health should conduct a Regulatory Impact Assessment (RIA) (with technical support from the Cabinet Secretariat) for better alignment of policy response and action to address the health, climate, gender, population dynamics, food security and environment nexus issues and associated implications at community, local and national levels. Strengthening multi-sectoral coordination is essential to harmonize policies across climate, health, gender, and development sectors, ensuring that implementation frameworks are cohesive and cross-cutting. The coordination unit can be housed under the Office of the Prime Minister, which should also be responsible for collecting and analyzing disaggregated data across sectors, including health, agriculture, education, and climate resilience, through structured surveys, community-based monitoring, and digital data platforms. Additionally, legal and policy reforms must reinforce protections against exploitation and gender-based violence, recognizing the heightened risks faced by women and girls due to climate-induced vulnerabilities. Gender-sensitive economic programs that promote financial inclusion, access to credit, and entrepreneurship in climate-affected regions should be prioritized.

At the local level, there is a need to build institutional capacity and strengthen community engagement to enable bridging policy gaps and ensuring sustainable implementation. Targeted training for policymakers, SRH practitioners, and climate adaptation stakeholders will deepen their understanding of the interlinkages between climate change, gender, and reproductive health, leading to more integrated and effective interventions. Engaging women, youth, and marginalized communities in decision-making processes will also be crucial in ensuring that policy responses are inclusive and reflect diverse needs. Moreover, expanding community-based SRH services in climate-vulnerable areas is critical to ensuring continued access to reproductive healthcare, particularly during climate-related disasters.

## Data Availability

The raw data supporting the conclusions of this article will be made available by the authors, without undue reservation. To protect the confidentiality of the participants, we will only provide raw data that does not include the names of respondents.
